# How Do Inflammatory Mediators, Immune Response and Air Pollution Contribute to COVID-19 Disease Severity? A Lesson to Learn

**DOI:** 10.3390/life11030182

**Published:** 2021-02-25

**Authors:** Cinzia Signorini, Patrizia Pignatti, Teresa Coccini

**Affiliations:** 1 Department of Molecular and Developmental Medicine, University of Siena, Via Aldo Moro, 53100 Siena, Italy; 2 Allergy and Immunology Unit, Istituti Clinici Scientifici Maugeri IRCCS, 27100 Pavia, Italy; patrizia.pignatti@icsmaugeri.it; 3 Laboratory of Clinical and Experimental Toxicology, Pavia Poison Centre, National Toxicology Information Centre, Toxicology Unit, Istituti Clinici Scientifici Maugeri IRCCS, 27100 Pavia, Italy; teresa.coccini@icsmaugeri.it

**Keywords:** COVID-19, SARS-CoV2, cytokine storm, genetic polymorphism, immune response, inflammation, pollution

## Abstract

Inflammatory and immune processes are defensive mechanisms that aim to remove harmful agents. As a response to infections, inflammation and immune response contribute to the pathophysiological mechanisms of diseases. Coronavirus disease 2019 (COVID-19), whose underlying mechanisms remain not fully elucidated, has posed new challenges for the knowledge of pathophysiology. Chiefly, the inflammatory process and immune response appear to be unique features of COVID-19 that result in developing a hyper-inflammatory syndrome, and air pollution, the world’s largest health risk factor, may partly explain the behaviour and fate of COVID-19. Understanding the mechanisms involved in the progression of COVID-19 is of fundamental importance in order to avoid the late stage of the disease, associated with a poor prognosis. Here, the role of the inflammatory and immune mediators in COVID-19 pathophysiology is discussed.

## 1. Introduction

The pathogenesis of coronavirus disease 2019 (COVID-19), which is due to infection by the novel coronavirus (SARS-CoV-2) [1,2], is driven by immune responses, hyper-inflammation, and hyper-coagulation [3,4,5]. COVID-19 resembles flu by involving the upper airways with mild symptoms, but patients may also develop severe symptoms with the involvement of the lower airways. Mainly, COVID-19 gives rise to a peculiar severe acute respiratory syndrome and bloodstream alterations as consequences of changes in inflammatory activities, which occur as defence mechanisms, leading to multi-organ dysfunction in patients at high risk [6].

SARS-CoV-2 targets host cells through the viral structural spike protein that binds to the angiotensin-converting enzyme 2 (ACE2) receptor. The spike protein is most prominent on the virion surface and confers the specific appearance of a corona [7]. The type 2 transmembrane serine protease (TMPRSS2), present in the host cell, promotes viral uptake by cleaving ACE2 and activating the SARS-CoV-2 S protein, which mediates coronavirus entry into host cells. Interestingly, ACE2 and TMPRSS2 are expressed in host target cells, particularly alveolar epithelial type II cells [8,9,10]. From the beginning of the pandemic, accumulated clinical experiences have shown that the prognosis of the disease was greatly influenced by the development of lung injury, which can evolve into the manifestation of acute respiratory distress syndrome (ARDS) that usually occurs in the late stages of the infectious process [1,11]. ARDS, whose pathophysiology is linked to inflammation and a dysregulated host immune response in the lung, results in an alteration of alveolar–capillary membrane permeability and tissue repair, leading to interstitial and alveolar oedema, which strongly impairs gas exchange. Therefore, ARDS is characterised by acute onset of hypoxemia and lung inflammation that evolves into multiple organ dysfunction syndrome and refractory hypoxemia [12,13]. Nevertheless, it has been discussed that COVID-19 pneumonia appears to be a specific disease with peculiar phenotypes, although the ARDS definition’s typical features are met [14]. Moreover, the phase of clinical worsening of respiratory functionality does not appear to be a manifestation and consequence of viral load peak but rather is the result of an exacerbation of the inflammatory-immune process [15,16,17]. Thus, in COVID-19, there would be a first phase in which the damage to lung epithelial cells is mediated directly by the action of the virus, which, like influenza viruses, causes cytotoxicity, and a late phase in which pulmonary damage is triggered by the immune response and inflammatory mediators. Therefore, the progression of COVID-19 has been divided into three main (i.e., viral, pulmonary, and inflammatory) stages [18].

The pathogenesis of COVID-19-induced pneumonia was described as very closely resembling autoimmune/auto-inflammatory syndromes [15,19,20].

The evolution and clinical complications of COVID-19 are greatly supported by the inflammatory process and the pathogenic activity of SARS-CoV-2. Thus, the most effective therapeutic treatments have been proven to be the same, or belonging to the same category, as those used in the therapeutic management of autoimmune diseases. As it is well known, these arise from defective or inappropriate immune responses but are sustained and complicated in their progression by degenerative processes moved by chronic inflammatory events. Therefore, in autoimmune disorders, the main pharmacological practices target inflammatory cytokines and their intracellular signalling pathways [21]. Accordingly, trials to assess the efficacy of anti-rheumatic therapies, such as hydroxychloroquine, and anti-cytokine therapies, such as interleukin (IL)-6 inhibitors for improving outcomes in COVID-19 patients are still ongoing [22,23,24,25,26]. In addition, a preliminary report on the use of dexamethasone in hospitalised patients with COVID-19 has been published [27].

Overall, the pathophysiological mechanisms that define the severity of COVID-19 can manifest with a very broad spectrum of phenotypic presentations ranging from an asymptomatic condition to a critical clinical condition, passing through intermediate symptoms [28].

Reflecting on inflammatory events, how surprising is the manifestation of the COVID-19 syndrome? To what extent do the mechanisms, reported as driving the disease, confirm the interrelationships between inflammation and immune response? How useful is the current knowledge of the mechanisms of the inflammatory process to improve the management of COVID-19 progression?

In COVID-19, the role of the inflammatory and immune mediators, together with genetic predisposition related to immune regulation, and exposure to air pollution, appear to be key factors for the disease severity.

## 2. Inflammatory Mediators in the Pathophysiology of COVID-19

The presence of an inflammatory stimulus triggers the production of a network of mediators, which by activating each other, are able to ensure a powerful and effective defence [29]. After this acute phase, usually, an anti-inflammatory response is mounted to limit dangerous events. Dysregulation of the inflammatory response is associated with many diseases such as asthma, cancer, atherosclerosis, diabetes, and autoimmune/degenerative diseases, including the so-called inflammatory diseases [30]. In this regard, inflammation is also involved in the pathophysiology of ARDS, leading to alveolar oedema and hypoxemia [12,31].

### 2.1. Hypoxemia

In COVID-19, hypoxia is a consequence of respiratory and circulation failure [32]. Hypoxia induces the hypoxia-inducible factor (HIF)-1α transcriptional activity [33], which regulate the cellular response to hypoxia, ensuring optimal functional, metabolic, and vascular adaptation to O_2_ deficiency [34]. Severe hypoxemia is a relevant feature of respiratory failure and ARDS, where the core of clinical management is mechanical ventilation [35]. As an adaptive response to tissue hypoxia, a consequence of persistent hypoxemia, oxygen sensing mechanisms work to increase breathing, heart rate, and blood flow. Accordingly, under hypoxic conditions, HIF is no longer degraded by prolyl hydroxylase (PHD) activity, and the PHD/HIF axis is a critically important oxygen-sensing pathway that mediates tissue adaptation to low oxygen environments [36]. Nevertheless, the PHD–HIF axis affects inflammatory processes, and cross-talk between hypoxia and inflammation has been identified [37]. HIF-1α increases macrophage aggregation, invasion, and motility and drives the expression of proinflammatory cytokines. Furthermore, HIF-1α increases neutrophil survival by inhibiting apoptosis and triggers the nuclear factor (NF)-κB-dependent neutrophilic inflammation [34]. Accordingly, the development of inflammation in response to hypoxia is clinically relevant, and in a wide array of human hypoxia-elicited inflammation, diseases have been reported [38].

In COVID-19 pathophysiology, where respiratory failure is one of the most relevant clinical features [39], hypoxemia represents a link to inflammation. Pathological mechanisms and disease outcomes have been proposed that involve hypoxia and HIF-1α-dependent detrimental cell signalling pathways. In severe cases of COVID-19, HIF-1α activation can lead to a cytokine storm (described below) by activation and stabilisation of immune cells including macrophages and neutrophils, causing the production of high amounts of inflammatory cytokines, vascular leakage (by up-regulation of the VEGF) and destruction of the alveolar-interstitial-endothelial epithelial complex barriers [40]. In particular, hypoxia, via innate immune cells, would induce cell death with a consequent increase in HIF transcription and cytokine release, whereas, in endothelial cells, hypoxia would induce HIF stabilisation, increase in VEGF, integrin levels, and vascular permeability. Finally, hypoxia would activate macrophages and neutrophils that contribute to ARDS and pneumonia [32]. In addition, it has been suggested that, under SARS-CoV-2 infection, chronic hypoxia may activate HIF-1α-driven pathways, leading to an increase in signalling mediated by TNFα, a molecule intimately involved in cytokine storms [32]. On the contrary, it has been speculated that, as a counteracting mechanism, the HIF-1α signalling pathway could decrease ACE2 and transmembrane protease serine 2 (TMPRSS2) and increase disintegrin and metalloproteinase domain-containing protein 17 (ADAM17) levels on the surface of alveolocytes and therefore decrease the invasiveness of SARS-CoV-2 [32].

HIF-1α has also implicated in the regulation of high levels of interferon (IFN) I [40], which is the main factor in the antiviral response of the innate immune system. IFN-I binding to its receptor triggers the Janus kinase-signal transducers and activators of transcription (JAK-STAT) pathway and regulates the expression of some important genes, such as protein kinase R (PKR), which are involved in viral component elimination from infected cells [40]. Induction of either IFN-β or IFN-γ upon SARS-CoV-2 infection results in activation of aryl hydrocarbon receptor (AhR) signalling leading to transcriptional upregulation of the expression of mucins in alveolar epithelial cells. Consequently, accumulated alveolar mucus affects the blood-gas barrier, thus inducing hypoxia and diminishing lung function [41].

Worthy of note, hypoxemia-induced expression of HIF may also activate platelets and coagulation factors. Consequently, the increase in tissue factor and plasminogen activating inhibitor-1 and inhibition of the endogenous anticoagulant protein S would occur [42]. Thus, in COVID-19, a hyper-coagulable state occurs due to the induction of a hyper-inflammatory state, activation of coagulation, and also downregulation of the ACE2/Angiotensin-(1-7)/Mas1 receptor axis (involved in increasing vasoconstriction) [4].

### 2.2. Neutrophils

Infection by SARS-CoV-2 triggers a local immune response, including recruitment of cell populations involved in innate immune response, which supports the inflammatory process, and the generation of viral-specific adaptive responses by both B and T cells, resulting in effective serum antibody titers as antiviral immunoglobulin-M and -G (IgM, IgG). Frequently, an appropriate immune response is mediated by antibodies, which bind and opsonise SARS-CoV-2, whereas alveolar macrophages phagocytise the neutralised viral particles. Additionally, the generation of viral-induced T cell responses eliminates infected cells and prevents cell-to-cell viral spread, reducing inflammation and lung damage. However, when the cytopathic effect of SARS-CoV-2 overwhelms the first line of defence, the innate immune response, alterations in signals regulating inflammatory homeostasis, release of damage-associated molecular patterns (DAMPs), and pathogen-associated molecular patterns (PAMPs) [43]. Such events contribute, together with the viral infection, to worse outcomes via an exacerbation of the inflammatory process [44]. The involvement of neutrophils in the inflammatory mechanisms of COVID-19 has been confirmed by an increased number of circulating neutrophils that have been reported to be an indicator of the severity of respiratory symptoms and poor clinical outcomes in COVID-19 [45]. The role of inflammatory signals in COVID-19 pathophysiology has also been confirmed by the increased concentration of neutrophil extracellular traps (NETs) detected in plasma, tracheal aspirate, and lung autopsies tissues from COVID-19 patients [46]. Since NETs are important mediators of tissue damage in inflammatory diseases, their release by healthy neutrophils, as a consequence of SARS-CoV-2 infection, contributes to the lung epithelial injury [47]. Interestingly, the release of NETs by healthy neutrophils is linked to the ACE2 receptor-serine protease axis, which is involved in the molecular interaction between the virus membrane glycoprotein spike (S) and human host cells [48], as previously mentioned. Nevertheless, peripheral blood neutrophilia has been found in COVID-19 [49,50]. Therefore, neutrophil-linked inflammatory mechanisms involved in COVID-19 pathophysiology rely on different conditions: (i) inflammatory mediators stimulate neutrophil activity and increase the trafficking of neutrophils to sites of inflammation; (ii) aberrant NET formation is linked to pulmonary diseases, thrombosis, mucous secretions in the airways, and cytokine production; (iii) neutrophils, able of phagocytosis, are implicated in the hyper-inflammation that drives to severe COVID-19. Their aberrant activation is linked to lung inflammation and elevated serum proinflammatory cytokines resulting in lung damage and thrombosis.

### 2.3. Cytokine Storm

Cytokines are part of the physiologically-regulated immune response to infectious agents, and the term “cytokine storm” was firstly described in the graft-versus-host disease [51] and as a serious adverse event developed after chimeric antigen receptor (CAR) T-cell therapy [52]. Currently, pathophysiological and clinical features of cytokine storms have been deeply described [53], and the term “cytokine storm” is used to describe a sudden cytokine release, which takes place when the immune system is over-activated with elevated levels of circulating cytokines and immune cells. Such hyper-inflammation and overproduction of inflammatory cytokines can be due to different conditions leading to a failure of negative feedback mechanisms meant to regulate the system [52]. Consequently, cytokine release syndrome occurs as a condition in which the immune response to the pathogen, but not the pathogen itself, can contribute to multiorgan dysfunction. The occurrence of a cytokine storm is associated with different clinical conditions, such as sepsis [54]. Cytokine storm was previously noted in SARS patients as an interferon-γ-related biological manifestation; it was described to be induced post-SARS coronavirus infection and was involved in causing immunopathological damage [17]. In COVID-19, cytokine storm has been defined among the primary pathophysiologic features [32], and it appears to be related to the involvement of virus interaction with ACE2. The consequent loss of control over angiotensin II activity, in addition to manifesting its effects on the altered homeostasis of arterial and/or pulmonary pressure [55,56,57,58], culminating in a hypertension status, activates cells of the immune system in lung tissue [15]. When COVID-19 progresses to severe illness, an immune system overreaction that culminates in abnormally increased serum levels of CCL2, CCL3, and CXCL10, has been reported. In patients with cytokine storm secondary to COVID-19, elevated serum cytokine levels are mainly related to IL-1β, IL-6, CXCL10, TNF-α, IFN-γ, macrophage inflammatory protein (MIP) 1α and 1β, and VEGF [1,59]. Levels of four cytokines (IL-6, CXCL8, TNF-α, and IL-1β) were associated with indices of severity concerning inflammation and coagulation: C-reactive protein (CRP), D-dimer, and ferritin. Moreover, IL-6 and IL-1β were additionally associated with fever [60]. Hugh serum levels were also detected for IL-17, G-CSF, GM-CSF, MCP1, MIP-1α in patients with severe COVID-19 [44]. Cytokine storm can lead to multi-organ failure [6,61]. Accordingly, IL-6 and IL-10 serum levels have been shown as disease severity predictors [62]. In particular, higher IL-6 levels were strongly associated with shorter survival [60]. Moreover, thromboembolic events (described below) appear to be more frequent in COVID-19 associated cytokine storm [63]. Thus, as a relevant point, the profile of serum cytokines is a predictor of the severity of disease [62]. Inflammatory cytokines may activate T-helper type 1 cell response as well as T-helper type 2-derived cytokines in COVID-19 patients [1]. Moreover, a further key event leading to hyper-inflammation seems to be the activation of an immune response regulated by T helper 17 (Th-17) lymphocytes [18]. Activation of Th-17 plays a role in neutrophil production and recruitment in COVID-19-associated cytokine storm [64].

Nevertheless, the role of cytokine storms in COVID-19-induced organ dysfunction is still under discussion [65]. A meta-analysis of data from 25 COVID-19 studies showed that inflammatory cytokine elevations in patients with severe and critical COVID-19, were lower than those reported in patients with other inflammatory syndromes, such as pulmonary disease unrelated to COVID-19, sepsis, and CAR T cell-induced cytokine release syndrome [65]. Additionally, acute-phase reactants (i.e., CRP, D-dimer, and ferritin) appeared to be similarly elevated in patients with COVID-19 and in patients with other inflammatory syndromes [65]. Sinha et al. [66] also critically evaluated the relevance of cytokine storm in COVID-19, showing that IL-6 levels in patients with COVID-19 (over 900 subjects) were above the normal range in many (but not all) cases. Nevertheless, the median values of IL-6 were lower than those typically reported in ARDS (over 1000 subjects).

From a therapeutic point of view, it should be considered that cytokines may be both detrimental, when they cause a cytokine storm, and essential when potentiating an antimicrobial response. Thus, blocking cytokine signalling through target therapies should be weighed depending on the risk of secondary infections and cytokine storm development [67,68].

### 2.4. Complement System

The complement system plays a central role in immunity and defence against pathogens [69]. Magnified complement activation contributes to the pathogenesis of many inflammatory and immune diseases [70], also including lung diseases induced by viral infections [71]. Although the complement system represents the first immune response to SARS-CoV-2 infection, there is growing evidence that unrestrained activation of complement induced by the virus plays a major role in acute and chronic inflammation [72]. In particular, the C5a complement factor, a chemoattractant involved in the recruitment of inflammatory cells, plays a key role in initiating and maintaining inflammatory responses by recruiting and activating neutrophils and monocytes, inducing the C5a-mediated neutrophil extracellular traps and the C5a-mediated cytokine storm [71]. Together with an increase in the amounts of plasma C-reactive protein (CRP), IL-6, and the chemokines CCL4 (macrophage inflammatory protein-1β), CCL2 (monocyte chemoattractant protein 1) and CXCL9 (monokine induced by interferon-γ), the levels of soluble C5a were increased according to the severity of COVID-19. In addition, high levels of C5aR1 receptor were found in blood and pulmonary myeloid cells [49]. It has been suggested that factors triggering the activation of the complement pathways are upregulated in COVID-19 and may sustain the high levels of C5a detected in patients with severe COVID-19 [49]. Furthermore, the involvement of C5a in the inflammatory process of COVID-19 is supported by identifing in patients with COVID-19, increased monocyte production of inflammatory cytokines, such as IL-6, TNF-α, and CCL2 [49]. Due to both the detection of C5a in the bronchoalveolar lavage fluid of COVID-19 patients with ARDS and pulmonary infiltration of macrophages largely expressing C5aR1, an association between C5a and lung disease caused by SARS-CoV-2 infection has been proposed [49]. Consistent with both such evidence and the high C5a levels described in preclinical models of acute lung diseases due to pathogenic viruses, including SARS-CoV, the C5a–C5aR1 axis has been thought as a target for a potential therapeutic strategy to treat severe COVID-19 [49]. Indeed, a promising therapeutic effect was observed when deteriorating patients were treated with anti-C5a monoclonal antibody [73]. Moreover, complement cascade was shown to be over-activated in lungs of COVID-19 patients, including C3 and C5b-9, deposition in type I and type II alveolar epithelial cells [73]. The complement C5b-9 and MAC deposition on tubules have been described to be involved in the acute renal failure associated with viral infection [74].

The complement system links innate immunity to coagulation [75]. Thus, aberrant activation of complement has been described to be involved in the pathophysiology of COVID-19 by promoting endothelial cell dysfunction, microvascular injury, and thrombotic events [76] and contributes to multiple organ failure [72]. Patients with severe COVID-19 infection often develop fulminant activation of coagulation reflected by thrombocytopenia, prolongation of the prothrombin time, and an elevation of D-dimer has been observed in these subjects [77]. In particular, terminal complement components C5b-9 (membrane attack complex, (MAC)) and C4d have been found in the microvasculature, and a co-localisation of COVID-19 spike glycoproteins with C4d and C5b-9 have been demonstrated in the interalveolar septa and the cutaneous microvasculature [76]. Thus, the complication of COVID-19 by venous thromboembolism occurrence appears to be primarily related to inflammatory-mediated mechanisms in addition to viral endothelium damage. Circulating cytokines, DAMPs, and PAMPs trigger blood monocytes to induce tissue factor (TF) expression. In these conditions, endothelial cells would take up viral particles and produce chemo-attractants that recruit monocytes and upregulate adhesion molecules. TF activates the extrinsic coagulation pathway leading to fibrin deposition and blood clotting [44]. Accordingly, thrombosis is very frequent in COVID-19, and frequent check of coagulation parameters is recommended [14].

### 2.5. Inflammasomes

Inflammasomes, are macromolecular inflammatory signalling complexes activated by the detection of pathogenic microorganisms and process pro-inflammatory cytokines (i.e., pro–IL-1β and pro-IL-18) to their bioactive forms [78], have been investigated in COVID-19. Activation of inflammasomes by SARS-CoV-2 infection is linked to COVID-19 disease severity and clinical outcomes [79]. Since activation of the NLRP3 inflammasome, a canonical component of inflammasomes acting as intracellular surveillance molecules [80], is relevant in different sepsis models [81], it has been speculated that NLRP3 would be linked to the pathogenesis of COVID-19 [79], whose pathophysiology is associated to a severe systemic inflammatory syndrome [82]. In particular, in lung tissues from lethal cases of COVID-19, active inflammasomes and CD14+ cells infected by SARS-CoV-2 and expressing NLRP3 were detected. Furthermore, COVID-19 patients were found to contain higher NLRP3 levels compared to control subjects [79]. By evaluating the inflammatory picture in COVID-19, it was reported that several inflammatory markers were correlated. In particular, (i) active/cleaved caspase-1 (Casp1p20) and/or cleaved IL-18, as indices of inflammasome activation, were positively associated with CRP, LDH, and ferritin; (ii) IL-18 levels were found to be positively correlated with IL-6 and CRP levels; (iii) and Casp1p20 positively correlated with IL-6, LDH, and CRP [79]. Moreover, it was found that the levels of IL-18 were higher in patients with body mass index ≥30 [79], suggesting a link between inflammasomes that activate IL-18 after viral infection and obesity.

With respect to the influence of inflammasome activation on the clinical outcome of COVID-19, it was found that (i) IL-18 levels, but not Casp1p20, were higher in patients who required mechanical ventilation; (ii) levels of Casp1p20 but not IL-18 were higher in patients with the severe form of COVID-19, and (iii) levels of IL-18, but not Casp1p20, were higher in lethal cases of COVID-19 when compared with survivors [79].

Thus, COVID-19 stokes inflammasomes and [83] the relevance of NLRP3 inflammasomes as a potential therapeutic target to manage clinical manifestation of COVID-19 have been discussed [84].

### 2.6. Inflammation in Extrapulmonary Manifestations of COVID-19

The extra-pulmonary effects of SARS-CoV-2 infection [44], also supported by inflammatory events, are well justified, given that ACE2 receptors are present in tissues different from the respiratory epithelium and lung parenchyma, such as myocardium, endothelium, and intestinal mucosa [85,86]. As compared to previously identified coronaviruses, SARS-CoV-2 shows a higher invasive capacity because of the higher affinity of its spike protein to ACE2 receptors. [87]. The involvement of neurons in SARS-CoV-2 infection [87] appears confirmed by the neuromuscular manifestations, such as anosmia and hyposmia, symmetric neuropathy, and myositis, which have been described as secondary effects of COVID-19 [88]. Again, these are symptoms supported, in their pathogenic mechanisms, by inflammatory events. If myalgia is part of the acute phase reaction in systemic manifestations of the inflammatory processes, together with fever, and if viral infections can cause typical influenza cases with gastrointestinal symptoms, what appears to be peculiar in COVID-19 is the simultaneous presence of all inflammatory symptoms or the ability of SARS-CoV-2 to damage, and consequently develop inflammatory symptoms, in multiple areas. This broad spectrum of symptoms is most likely due to the widespread distribution of ACE2 receptors. Interestingly, the presence of ACE2 receptors in the brain makes it necessary to pay attention to cerebral microcirculation. Additionally, neuronal inflammation should be checked during the therapeutic management of COVID-19 [89].

**Figure 1:**
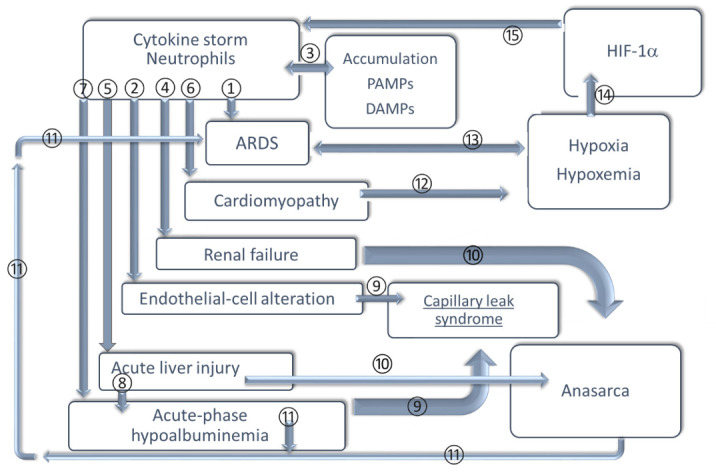
A picture of coronavirus disease 2019 (COVID-19) pathophysiology triggered by the inflammatory response. ① Neutrophils are predominate in pulmonary oedema fluid in acute respiratory distress syndrome (ARDS). These cells adhere to endothelial cells through the interactions of leukocyte integrins and intercellular adhesion molecule (ICAM) located on the endothelial surface, contributing to pulmonary neutrophil sequestration [12].② These cells release toxic oxygen radicals, proteases, cytokines and products of arachidonic acid metabolism, damaging endothelial cells with increased vascular permeability. ③ In the event of alterations in signals regulating inflammatory homeostasis (due to viral cytopathic effect that overwhelms the first line of the innate immune response), and an accumulation of DAMPs and PAMPs occurs [43], contributing to exacerbation of the inflammatory process [44]. ④⑤⑥ In severe cytokine storm cases, renal failure, acute liver injury, and cardiomyopathy can develop [53]. ⑦⑧ Hypoalbuminemia occurs in COVID-19 cytokine storm [53]. It is also a consequence of liver injury (reduced hepatic albumin synthesis). ⑨⑩ The combination of endothelial-cell alteration and acute-phase hypoalbuminemia renal dysfunction [90] can lead to anasarca and capillary leak syndrome—a rare disorder characterised by a dysfunctional inflammatory response, endothelial dysfunction, extravasation of fluid, hypoalbuminemia, and subsequent organ failure [53]. ⑪ Hypoalbuminemia is a known factor in ARDS [90], a clinical syndrome of non-cardiogenic pulmonary oedema that in the exudative stage is characterised by an increase in vascular permeability and alveolar oedema [12]. ⑫ Cardiomyopathy causes hypoxia and hypoxemia. ⑬⑭⑮ Hypoxia activates macrophages and neutrophils that contribute to ARDS and pneumonia [32] and stabilise HIF-1α leading to cytokine storm by activation of immune cells [40]. Abbreviations: DAMPs, damage-associated molecular patterns; HIF-1α, hypoxia inducible factor 1α; PAMPs, pathogen-associated molecular patterns.

An additional feature of COVID-19, which could be implicated in the multiplicity of organ targets and systemic symptoms, concerns a supposed interaction between SARS-CoV-2 virions and plasma albumin reflecting depletion of the endothelial glycocalyx layer [90]. Such alteration modifying endothelium integrity would undoubtedly refer to pathophysiological aspects of the blood circulation that manifest themselves in COVID-19 and complicate the clinical course of the disease. Accordingly, hypoalbuminemia is a relevant factor in sepsis; ARDS, which is associated with sepsis, is systemic and is associated with maintenance of circulation homeostasis. A summary of the complex interrelationship among inflammatory factors in COVID-19 is displayed in Figure 1.

## 3. Inflammation and Immune Response—Molecular Cross-Talk

Viral infections are sometimes responsible for concomitant intestinal and upper respiratory airway pathological manifestations, and cooling and conjunctivitis symptoms are also possible [7,91]. Both coronaviruses causing SARS in humans, SARS-CoV-1 and SARS-CoV-2, share the ACE2 cellular receptor interaction with the spike glycoprotein as a means of cellular entry and are equally stable and infectious as aerosols [7].

During the pandemic, what is surprising and has put health management in an emergency condition are the extent of the symptoms, which can easily progressively worsen and become complicated, and the high contagiousness of SARS-CoV-2.

Usually, coronaviruses interact with human biological systems in a transient and mild manner. The modality of interactions of SARS-CoV-2 with human cells may rely on SARS-CoV-2′s ability to evade the immune defences, similar to what neoplastic cells can do, which reduces the activity of the immune response by modifying the interactions and activations of white blood cells. This obstacle for the host’s defensive response creates a useful and sufficient window of time within tumour cells multiply easily. Likely, SARS-CoV-2 viral particles might behave similarly. Beyond this limited time window, the interaction between host and etiological agent advances, and the immune defensive response requires more complex B and T cell mechanisms to be efficient. Accordingly, a reduction in both T cells and natural killer (NK) cells is a relevant feature of COVID-19 and is linked to the disease severity [92,93,94,95]. The therapeutic advantages resulting from the use of antibodies capable of hindering the infection leads us to think about B cells’ prevailing role compared to cytotoxic immune mechanisms. In addition, it has been discussed the stability of the immunological memory raised by SARS-CoV-2. It has been hypothesised that a better-preserved immunological memory, and acquired after common cold coronavirus infection, could represent the factor highly responsible for a better prognosis, or even absence of relevant clinical symptoms, in children and young people as compared to older. Compatibly, acute SARS-CoV-2 infection leaves activated T cells and specific antibody responses [96]. Is this the first time that a coronavirus causes different symptoms in subjects of different ages? The clinical experiences accumulated during the pandemic period indicate that the negative influence of age on the effectiveness of inflammatory and immune processes should not be underestimated, in the same way that the incidence of neoplasms increases with age due to defects and slowdown of cell repair processes. A further item to be considered is immunosenescence, which represents age-related variation of immune responses [97]. In particular, in older people, a decrease in naïve T cells in favour of an increase in terminally differentiated T cells is observed. Furthermore, the ratio between pro-inflammatory Th-17, in which its role appears to be relevant in COVID-19 pathophysiology as reported above, and the anti-inflammatory regulatory T cells, is disturbed during ageing [98]. As an outcome, the susceptibility to infections could be increased [99]. It is worth noting that the observation of symptoms with different entity has been made for SARS-CoV infection, and studies on mice revealed one of the possible molecular mechanisms, which is the increase in the activity of anti-inflammatory factors: phospholipase A_2_ group IID (PLA_2_G2D) in the lungs of older subjects [100].

In the inflammatory response during COVID-19 [101], inflammaging could be invoked as a link between increased susceptibility to COVID-19 and advancing age. Inflammaging appears to be the result of over-stimulation of inflammation due to the accumulation of alarms due to degenerative events [102]. Therefore, if, on the one hand, the proinflammatory phenotype of senescent cells helps the protection from infectious diseases, at the same time, this reduced threshold of activation of inflammation facilitates the achievement of cytokine storm [103].

The defence mechanisms represented by inflammation and the activity of immune cells, even when involved in hindering a virus that is the causative agent of colds, become part of precision medicine, where prevention, diagnosis, prognosis, and treatment are focused on the target subject. Precision medicine is a part of healthcare that considers individual variability and represents an ever-developing medical approach [104,105]. In this matter, the ABO blood group system has been reported to be relevant in defining susceptibility to COVID-19 [106]. Such a relationship between red blood cell antigen profiles and susceptibility to infections could be linked to the cross-talk between erythrocytes and the immune system. The role of red blood cells in co-stimulating the T cell proliferation [107] and enhancing B cell responses to antigens [108] have been reported. In addition, the function of erythrocytes in modulating the role of dendritic cells in inflammatory processes has also been described [109,110].

The fact that serum therapy could be clinically advantageous and decisive in the initial stages of COVID-19 confirms that the late stages of the disease are not supported by the infection and action of SARS-CoV-2 but that, almost prevalently, they are due to the deregulation of the inflammatory process. Such short-circuit of the inflammatory mechanisms involves a self-sustaining and self-aggravation of inflammation, similar to chronic inflammatory events or, more properly regarding COVID-19, to shock conditions.

## 4. What Makes the Difference between Asymptomatic and Mild/Severe Symptomatic Infected Patients?

From the beginning of the spread of SARS-CoV-2 infection, it was clear that people responded to the infection in different ways, from asymptomatic condition to severe multiple organ symptoms. From the very beginning, the presence of comorbidities was recognised as giving an increased risk of developing severe COVID-19, particularly for cardiovascular diseases, diabetes, and obesity [111,112,113,114]. Many reviews have been published on COVID-19 and comorbidities. Hypertension is one of the most frequent comorbidities present in COVID-19 patients [115]. The renin-angiotensin system (RAS) has a pivotal role in both the pathophysiology of hypertension and the SARS-CoV-2 infection since the ACE2 receptor is the virus’s main access mechanism to enter human cells [116]. Therefore, a strict relationship between virus infection and hypertension was predictable. Furthermore, hypertension has been demonstrated to be associated with immune system activation, as shown by high IgG serum levels and increased CD8 T cells able to produce IFN-γ, TNF-α and Th17 cells [117]. All these conditions favour severe COVID-19 symptoms. Regarding diabetes, susceptibility to SARS-CoV-2 infection seems equal to the general population [118], but diabetic patients, once infected, have a higher risk of developing severe symptoms. Mechanisms involved in this negative progression are mainly: (a) higher expression of ACE2 in the lung, increasing the likelihood of spreading of the virus [119]; (b) unbalanced ACE2/ACE ratios with consequent increased inflammatory and oxidative stress responses [120]; (c) high glucose concentrations in the fluid lining the lungs favouring viral replication [121]; (d) endothelial dysfunction with higher pro-thrombotic risk [122]; (e) higher predisposition of developing respiratory infections [123]. Obesity has been reported as highly prevalent in COVID-19 patients, and mechanisms under evaluation are: (a) an imbalance in the RAS; (b) low grade of systemic inflammation present in obese patients, which could favour the development of a cytokine storm; (c) frequent association of obesity with hypertension and diabetes; (d) higher viral shedding in obese patients already reported for the influenza virus; (e) higher leptin and lower adiponectin levels leading to unbalance between anti- and pro-inflammatory mechanisms; (f) altered pulmonary functions in obese patients, which can favour the progression of COVID-19 and severe symptoms [124].

Increased age is certainly a risk factor due to the frailty of old people and to the presence of these comorbidities. However, young people with a severe form of COVID-19 and older asymptomatic subjects have been reported. In preliminary studies from China, in a small number of subjects, asymptomatic patients were younger, more frequently women, and with a BMI < 25 [125]. Blood cells, cytokine levels and T lymphocytes have been reported as significant predictors for asymptomatic infected people [126].

It has been reported that blood groups could influence susceptibility to the virus [127]. Zhao et al. analysed 2173 COVID-19 patients and a huge amount of non-COVID-19 subjects in the Wuhan region of China.They found a significantly high risk for COVID-19 in blood group A subjects and a significantly low risk of infection in blood group O subjects [127]. The same distribution of the risk was confirmed in deceased patients. The cause of reduced susceptibility in group O subjects could be the presence, in these subjects, of natural antibodies to group A which can inhibit the adhesion of the virus to the ACE2 receptor as demonstrated in vitro for SARS-CoV in cell lines expressing ACE2R [128]. These data were subsequently confirmed by the study of Zietz et al. [129] in COVID-19 patients with different levels of severity; whether ABO groups could have a role in asymptomatic COVID-19 remains to be elucidated.

Using pedigree and population strategies, an insertion destroying the transcription of the gene for dipeptidyl-peptidase 7 (DPP7), an innate immunity response enzyme, was found in two asymptomatic subjects [130]. Furthermore, the missense variant rs12329760 in the *TMPRSS2* gene, which is involved in the activation of SARS-CoV and SARS-CoV-2 proteins, was found to be less frequent in severe patients [130]. Between severe and less severe patients, the involvement of class I rather than class II of *HLA* genes was identified. An increase in frequencies of HLA-A*11:01, B*51:01, and C*14:02 alleles was found in severe patients compared to mild-moderate, while HLA-B*46:01 linked to SARS-CoV infection was not found to be associated with disease severity in COVID-19 patients [130].

Urbach et al., from the results of an online symptom-tracking survey administered to 3654 subjects, found that statin use was associated with a lower risk of developing symptomatic COVID-19 [131] when symptoms and/or RT-PCR were used to select patients. Other studies had previously reported the association between statins and low or absent COVID-19 symptoms [132,133] or decreased mortality [134]. Different hypotheses on the relationship between statin intake and mild COVID-19 symptoms have been proposed. Inhibition of the main protease of the virus (Mpro) has been demonstrated in silico by some statins suggesting a possible reduction in virus spreading in subjects taking these drugs [135]. Another hypothesis could be independent of COVID-19 infection but more related to the already reported alleviation of flu-like symptoms and pneumonia by statins [136,137]. In another study, COVID-19 patients, both asymptomatic and severely ill, aged 30–60 years, were prospectively followed for six weeks and blood biomarkers evaluated. Serum vitamin D levels were significantly higher in the asymptomatic group, and the prevalence of vitamin D deficiency (<20 ng/mL) significantly higher in subjects with severe symptoms [138]. Although the study was carried out in India, where Vitamin D deficiency is not unusual, and other possible confounding factors were not taken into account, considering the numerous effects of Vitamin D on the immune system [139], its relationship with COVID-19 symptoms should be better analysed.

Virus infections, SARS-CoV-2 included, generate increased pro-oxidant processes, partly triggered by TNF-α production. This rise can be expressed by serum ferritin increase, thrombocytopenia, lymphopenia, haemolysis with an increase in serum LDH levels. The production of reactive oxygen species (ROS) is part of the natural response to infections and is self-limited by anti-oxidant processes with a pivotal role for nitric oxide (NO) and glutathione (GSH). Production of these compounds is dependent on nicotinamide adenine dinucleotide phosphate hydrogen (NADPH) produced principally by glucose-6-phosphate dehydrogenase (G6PD) [140]. Among the several hypotheses regarding predisposition to develop severe symptoms during COVID-19, G6PD deficit has been considered. Italy and Spain, two of the European countries mostly involved during the first spread of SARS-Cov-2, had a high frequency of inherited G6PD deficits. However, preliminary studies did not confirm an association between COVID-19 and this deficit [130] or differences between asymptomatic and symptomatic infected patients [141], even if a G6PD deficit was present in more than 30% of infected subjects. G6PD deficits can be acquired particularly in patients with metabolic syndrome [142]. Therefore, Buinitskaya et al. [140] hypothesised that a G6PD deficit caused by metabolic syndrome could be responsible for decreased NO and GSH reducing the capacity to counteract SARS-CoV-2 and increasing multi-organ damage by reactive oxygen species (ROS).

The type and intensity of the immune response have been considered in order to understand differences between asymptomatic and symptomatic subjects in COVID-19. A genome-wide association study (GWAS) found an association between the severity of the disease and a multi-gene cluster on chromosome 3. Among different genes, chemokine receptors of the C–C and CXC families are located in this cluster (e.g., *CXCR6* and *CCR9*) and are involved in the migration of T cells during cell activation [143]. Family studies have allowed the identification of a polymorphism in the coding region of Toll-like receptor 7 (*TLR7*) gene determining a loss of function associated with a reduced IFN I and II response after viral infection [144]. This polymorphism could be associated with an inability to inactivate the SARS-CoV-2 virus and switch towards a more dangerous and pronounced immune response [95]. Furthermore, the *TLR7* gene, together with other genes suspected to be involved in COVID-19, is located in the X chromosome, partly justifying the increased susceptibility of the disease in male found in some studies [143]. Considering the pivotal role of type I IFNs in antiviral response, a decrease in the activity of these factors could be due to the presence of autoantibodies against IFNs, as demonstrated by Bastrad et al. [145] and Hadjadj et al. [146]. They found that around 10% of patients with life-threatening COVID-19 (94% males) had neutralising autoantibodies against IFN-ω and IFN-α or both, associated with low or undetectable amount of these IFNs in their blood during the acute phase of the disease. These autoantibodies, already present before SARS-CoV2 infection and almost absent in asymptomatic patients and in uninfected controls, neutralised in vitro the ability of IFN-I to block SARS-CoV2 [145]. These data support the hypothesis that inborn errors of immunity could be at the basis of at least a part of severe COVID-19.

**Figure 2:**
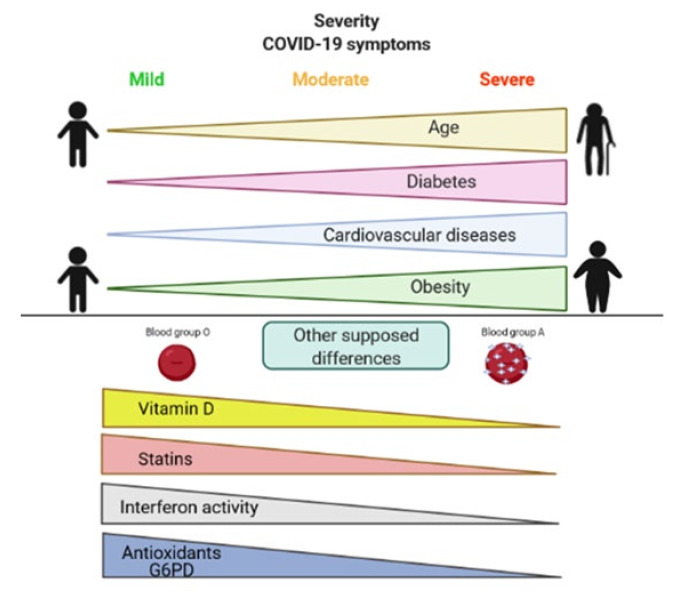
Clinical and biochemical features involved in disease symptom severity in SARS-CoV-2 infected subjects. Above, the role of primary factors of risk (clinical features: age, diabetes, cardiovascular diseases, and obesity [111,112,113,114,125,150]) for severe disease are shown. Below, other factors (biochemical features: levels of vitamin D, statins, interferon activity, and G6PD [132,133,134,138,140,145]) that are suggested to be involved in COVID-19 severity are summarised. Abbreviations: G6PD, glucose-6-phosphate dehydrogenase. The figure was created with Biorender.com.

Differences in limiting virus spread inside the body could be highlighted by differences in the viral load. In a study conducted in northern Nevada during the first SARS-Cov-2 wave, patients with more severe disease, including fatal cases, had a significantly higher viral load [147]. In another study, a progressive increase in viral load measured through cycle threshold correlated with disease severity [148]. Opposite results were obtained in a study performed in a limited number of subjects by Hasanoglu et al. [149], who found a significantly higher viral load in asymptomatic subjects with a negative correlation between viral load and age. Differences could be due to the time elapsed between the start of the viral infection and PCR tests, which is quite difficult to standardise. Furthermore, it has been reported that men had a longer duration of viral shedding which increases with age [150], suggesting that more data are needed to clarify the association between viral load and COVID-19 symptoms. A summary of differences between patients according to the severity of COVID-19 is displayed in Figure 2.

Once SARS-CoV-2 infection has begun, the humoral immune response is induced, and specific IgM and IgG are generally produced within two weeks. The production of antibodies might also differ between asymptomatic and symptomatic patients. In a study by Lei et al. [151], a huge amount of subjects was screened with both PCR analysis and serological evaluations among epidemiologically suspected individuals. Antibodies against SARS-CoV-2 can be directed to different proteins of the virus, and only some of these antibodies have the capability to neutralise the interaction between the virus and ACE2 receptors. The authors found that asymptomatic subjects mostly produced antibodies to proteins S1, part of the virus spikes, and N, part of the virus nucleocapsid [151]. Antibodies to nucleocapsid (N) protein seem to persist and be continuously produced while IgM to S1 protein peaked after 17–25 days of infection, and then in two months disappeared. More than one-third of asymptomatic patients did not produce neutralising antibodies, and when produced, they rapidly disappeared [151]. Other studies confirmed the lower production of antibodies, particularly the neutralising ones in asymptomatic subjects with a correlation between antibody titles and viral load [148]. Mild patients who did not need hospitalisation had a higher ratio of antibodies to the receptor-binding domain (RBD), or to the S1 domain, of spike than to the N protein, suggesting a higher capability to block virus invasion after infection [148]. Actually, we do not know whether the loss of specific antibody production a few months after a previous infection exposes people to re-infection as T cell immunity could anyway protect from the infection and memory T cells are mainly involved in long-term immune protection against microorganisms. Sekine et al. [152] demonstrated that after the acute phase of COVID-19, in which cytotoxic specific T cells were found, a pool of memory T cells was produced with a stem-cell-like phenotype. In this study, asymptomatic subjects and seronegative relatives of positive patients presented with SARS-CoV-2-specific T cells, even in the absence of serum specific antibodies, suggesting the ability of these subjects to elicit a functional T cell response against the virus [152]. The presence of memory T cells years after infection was previously reported also for Middle East Respiratory Syndrome (MERS) and SARS-CoV-1 infections [153,154].

Taking together these considerations, we can conclude that many factors could be responsible for the different patterns of response to SARS-CoV-2 infection, from asymptomatic to severe symptoms with death. Published studied highlighted only a few of these possibilities, and many others are under evaluation.

## 5. Air Pollutants as Stressors Contributing to Severe Consequences of Exposure to SARS-CoV2

In the frame of the dramatic global scenario of SARS-CoV-2 infection, in 2020, there was considerable growing evidence pertaining to factors that may contribute to the different degree of disease severity.

The most severe consequences from COVID-19 and influenza stem from a degraded/dysfunctional immune system and the exploitation of the degraded immune system by the virus.

In a healthy immune system, the virus would be unable to overcome its strong defences and would be neutralised; on the other hand, some people have an intrinsically dysfunctional immune system due to genetic/hereditary/congenital factors [155]. However, other factors may play a much stronger role in determining a successful immune response. Currently, the adverse impacts of several factors, such as toxic lifestyle, iatrogenic, biotoxic, environmental/occupational, and psychosocial/socioeconomic factors, on the health of the immune system directly or indirectly have been underlined in recent reviews [156,157]. Many of these factors that contribute to a degraded/dysfunctional immune system are pervasive; they contribute to myriad (especially chronic) diseases/non-communicable diseases (NCDs) [158,159]. Thus, people with an immune system degraded by the above contributing factors also have an increased likelihood of having significant comorbidities, such as those that make people the most vulnerable to succumbing to COVID-19.

With respect to environmental factors, a growing number of recent studies supports that ambient air pollution, characterised by a high population attributable fraction, plays a key role in increasing the likelihood of spread of SARS-CoV-2 and severe clinical outcomes in COVID-19 [157,160,161,162,163,164,165,166].

The impact of ambient air pollution on excess morbidity and mortality has been well established over the last several decades [167,168,169], and numerous epidemiological studies have shown the effects of air pollution on respiratory and cardiovascular systems in particular. In addition, air pollution has also been associated with multiple negative effects on the nervous system [170].

Both short-term and long-term studies on air pollution effects have given estimates of damage due to increasing noxious exposures, by using attributable population fractions or excess fractions as the metric of effects [171]. In particular, major ubiquitous ambient air pollutants, including fine particulate matter (PM) in size fraction PM_2.5_, nitrogen dioxide (NO_2_), and ozone (O_3_), have both a direct and an indirect systemic impact on the human body by enhancing oxidative stress, inflammation, and respiratory infection risk, eventually leading to dysfunction and deterioration on the respiratory, cardiovascular and immune systems [172,173,174,175,176], which are definitely involved in COVID-19. Actually, the COVID-19 pandemic has dramatically shown that infectious diseases and NCDs are highly interconnected. For the latter, risk factors, including chemical toxicants, air pollution, climate change and socio-economic determinants, strongly also contribute to the severity of the former [157,177]. It follows that the study of the interaction between viral infections and environmental factors of chronic diseases is fundamental for established efficient preventive health measures to develop better treatments adapted to co- and multi-morbidities, as well as doing so cost-efficiently.

Although the epidemiologic evidence is still limited, previous findings on the outbreak of SARS revealed a crude positive correlation between air pollution and SARS case-fatality rate in the Chinese population without adjustment for confounders [178]. Nevertheless, this was the first observation showing that air pollution is associated with the increased fatality of SARS patients in the Chinese population (Chinese SARS epidemic in 2003), and thereafter it was also utilised for comparison USA data in a recent epidemiological analysis [162] to evaluate the degree to which air pollution influences COVID-19 mortality. Epidemiological analysis of the first SARS-CoV-1 outcomes in 2003 [178], and the investigations of those for SARS-CoV-2 since 2019, provide evidence that the incidence and severity are related to ambient air pollution [162].

Several recent studies have analysed whether the different areas of the world with a high and rapid increase in COVID-19′s contagion were correlated to a greater level of air pollution, such as (i) high levels of air pollution over the last years, which made the population more sensitive to COVID-19 (long-term exposure); (ii) sensitivity to the virus, which was linked to the high level of air pollution in the period when the virus appeared (short-term exposure).

For example, a recent analysis of 213 cities in China demonstrated that temporal increases in COVID-19 cases were associated with short-term variations in ambient air pollution [179]. Another study confirmed a statistically significant relationship between short-term exposure to higher air pollutants, namely PM_2.5_, PM_10_, carbon monoxide, NO_2_, and O_3_, and an increased risk of COVID-19 infection in 120 cities in China between 23 January 2020 and 9 February 2020 [179]. This evidence was confirmed by different studies analysing the air quality in Italy and China in the period of maximum COVID-19 virulence [180,181].

With respect to the well-known contribution of the chronic exposure to atmospheric PM to increased hospitalisations and mortality, primarily affecting cardiovascular and respiratory systems and causing premature deaths estimated to be over two million per year worldwide, a recent review highlighted the potential role of PM in the spread of COVID-19 in particular. The study focused on Italian cities (e.g., Bergamo, Brescia, and Milano) where a high and rapid increase in COVID-19′s contagion occurred from March 2020 and where PM daily concentrations were found to be higher than the annual average allowed during the months preceding the epidemic [165].

Concerning the long-term exposure, statistically significant positive correlations were found between COVID-19 infections and high levels of air pollution in several countries such as China, Iran, Italy, Spain, France, Germany, the United Kingdom, and the USA. In Italy, the correspondence between poor air quality and COVID-19 appearance and its induced mortality was the starkest [182]. The positive correlations between SARS-CoV-2 infections and air quality variables in China, Italy and the USA indicated that higher mortality was correlated with high PM_2.5_, carbon monoxide, and NO_2_ values [183]. In Northern Italy, particularly affected by COVID-19, the population had been constantly exposed to a chronic high level of air pollution [184,185]. The conclusive data of these papers indicated that long-term air-quality significantly correlated with cases of COVID-19 in up to 71 Italian provinces, providing further evidence that long-term exposure to air pollution may represent a favourable context for the spread of the virus.

A study by Wu et al. [186] proved a positive correlation between COVID-19 mortality rates and long-term PM_2.5_ exposure using county-level data from the United States, showing that an increase of 1 μg/m^3^ in PM_2.5_ was associated with an 8% increase in the COVID-19 mortality rate.

Recent studies evaluating the correlation of NO_2_ and mortality rates in northern Italy [187,188] and regions of England [189] also demonstrated a direct relationship.

A cross-sectional, nationwide study in the United States estimated the association between long-term (2010–2016) county-level exposures to NO_2_, PM_2.5_, and O_3_ and county-level COVID-19 case-fatality and mortality rates in the USA, and indicated that long-term exposure to NO_2_, which largely arises from urban combustion sources such as traffic, may enhance the susceptibility to severe COVID-19 outcomes, independent of long-term PM_2.5_ and O_3_ exposure [160]. On the other hand, the impact of four ambient air pollutants on the COVID-19 mortality rate in the United States, examined by regression analysis, showed that ground-level O_3_ and NO_2_ concentrations might also contribute to a greater COVID-19 mortality rate [161].

Positive correlations between PM_2.5_ levels and the incidence, mortality rate, and case fatality rate of COVID-19 were also found in an Italian study evaluating 110 provinces during a period from 20 February to 31 March 2020. The results not only confirm the supposed link between air pollution and the rate and outcome of SARS-CoV-2 infection but even support the hypothesis that pollution-induced over-expression of ACE2 on human airways may favour SARS-CoV-2 infectivity [163].

Air pollution and fine particulate matter (PM_2.5_), as its main component, resulted as the most important predictors of SARS-CoV-2 effects in another Italian study with the help of artificial intelligence. The study indicated that the emissions from industries, farms, and road traffic—in order of importance—were the most air pollution sectors linked to an increase in mortality rates of the 20 Italian regions. Moreover, the road traffic resulted in the most important variable related to SARS-CoV-2 positivity. Given the major contribution played by air pollution (much more important than other health and socio-economic factors), the study also forecasted that, with an increase of 5–10% in air pollution, similar future pathogens may inflate the epidemic toll of Italy by 21–32% additional cases, whose 19–28% more positives on pathogen and 4–14% more deaths. The findings, demonstrating that fine-particulate (PM_2.5_) pollutant level is the most important factor to predict SARS-CoV-2 effects that would worsen even with a slight decrease in air quality [164].

In a study conducted in a small area of Catalonia, Spain, from 25 February to 16 May 2020, on the association between long-term exposure to air pollutants and increased risk of incidence and death from COVID-19, the authors showed that the long-term exposure to NO_2_ and to a lesser extent PM_10_ were independent predictors of the spatial spread of COVID-19. For every 1 μm/m^3^ above the mean the risk of a positive test case increased by 2.7% for NO_2_ and 3.0% for PM_10_. Regions with levels of NO_2_ exposure in the third and fourth quartile had 28.8% and 35.7% greater risk of a death, respectively, than regions located in the first two quartiles. Although the data support the existing of biological mechanisms that may partially explain the association between long-term exposure to air pollutants and COVID-19, the authors also hypothesise that the spatial spread of COVID-19 in Catalonia may be attributed to the different ease with which some people, the hosts of the virus, have infected others [166].

The degree to which air pollution, fine particulates specifically, influences COVID-19 mortality was also recently derived from epidemiological data in the USA and China. The study estimated that particulate air pollution contributed to 15% of COVID-19 mortality worldwide, 27% in East Asia, 19% in Europe, and 17% in North America. Globally, 50–60% of the attributable anthropogenic fraction was related to fossil fuel use, up to 70–80% in Europe, West Asia, and North America. These results add to evidence that air pollution is an important cofactor increasing the risk of mortality from COVID-19 and provide motivation for combining ambitious policies to reduce air pollution with measures to control the transmission of COVID-19 [162]. Table 1 summarises the main reported findings underling the relationship between high levels of air pollutants and increased risk/fatality of SARS-CoV-2 infection.

Notably, some authors also hypothesised that an atmosphere rich in air pollutants, together with certain climate conditions, may have promoted a longer permanence of the viral particles in the air, thus favouring an “indirect” diffusion [180,181], which thus may have played an important role in increasing the contagion [190]. In fact, the anomalous anticyclonic system over the western Mediterranean basin (centred between Spain and Italy during February 2020) and lower pressures over Northern Europe may have produced dry conditions over southwestern Europe, thus providing optimal meteorological conditions for virus propagation, both indoors and outdoors, in addition to the direct and indirect contact and short-range droplets.

ROS production induced as a consequence of the interaction with environmental air pollutants is pointed out as a critical mechanism that may predispose mainly elderly populations but does not exclude young subjects [185], even those presenting previous chronic conditions of lung inflammation or neuroinflammation, to the most serious consequences of COVID-19 [191]. It is well known that pollution impairs the first line of defence of the upper airways, namely the cilia functions of epithelial cells [192]; thus, a subject living in an area with high levels of pollution is more prone to develop chronic respiratory conditions and is more vulnerable to any infective agent.

Another important point to consider in this scenario is that the airborne particles, which constitute the main threat to human health, including the nanosized particle (1–100 nm) fraction. This latter is highly abundant in the urban atmosphere and has the ability to penetrate virtually all organs, and possesses high bioreactivity. These nanosized particles (NPs) have the potential of carrying toxic metals, spores, viruses, and bacteria. They have also been linked to respiratory viral infections such as the SARS-CoV-2 virus and influenza, as well as other respiratory and cardiovascular diseases. In recent years, science has found augmenting evidence that NPs generated by transport (e.g., fuel combustion, tire wear, and brake wear) cause not only adverse health effects to the respiratory and cardiovascular systems but also promote neurodevelopmental and cognitive impairment. Recent works have also underlined that exposure to NPs could predispose exposed populations to contracting viral infections in general and, more specifically, to contracting COVID-19-associated immune pathologies [193,194].

The SARS-CoV-2 virus induces neurological complications, and the possible long-term impact for neurological and especially neurodegenerative diseases can only be anticipated [195]. In a worst-case scenario, the common olfactory route of SARS-CoV-2 and NPs may exacerbate the adverse health effects also on the central nervous system. This evidence has been raised in a recent study on Metropolitan Mexico City, a city where the development of Alzheimer diseases starts in childhood, underlining the necessity to deeply investigate why the residents chronically exposed to air pollution are likely to be more susceptible to the systemic and brain effects of SARS-CoV-2 [194]. Essentially this paper describes very important social and clinical challenges related to behavioural, cognitive, and neurological manifestations in healthy as well as susceptible young people, and long-term exposure to NPs and SARS-CoV-2 infection. In particular, remarkable questions to be explored are how SARS-CoV-2 manage to enter the brain at ease, how NPs contribute to the process, what could be the entity of neuronal damage (e.g., is it synergistic?), and how may this influence the neurodegenerative process.

Altogether, these data underline, in countries like, for example, India, China, Italy and the USA, a positive correlation between the existing levels of higher air pollutants and severity/death rate under SARS-CoV-2 infection. Environmental factors may partly explain the behaviour and fate of COVID-19 [196]. For instance, air pollution acts as the causative agent for diseases such as bronchitis, asthma and many other respiratory diseases. The SARS-CoV-2 uses respiratory tracts as its primary attacking sites because of the predominant expression of ACE2 in their epithelial cells. The protein ACE2 acts as the receptor for the attachment of the SARS-CoV-2 spike protein S and hence increases the chance of infection as well as the severity of the disease in humans. The COVID-19 is known for its fatal activity by causing respiratory choke and, therefore, causes mainly respiratory disorders and common cold associated symptoms. Therefore, these observations indicate that COVID-19 and air pollution have intricate relations with each other. On the one hand, polluted air can create many breathing issues in humans leading to easy ways for the virus to enter and infect, and, on the other hand, NO_2_ and PMs, especially PM_2.5_, can be responsible for the over-expression of ACE2 in human respiratory cells increasing the risk of getting attachment of the virus through its interface spike protein S into the host epithelial cells along the respiratory tracts. As a result, the virulence of the virus in terms of efficient infection could be increased in areas where air pollution is high. This could increase the risk of infection as well as the facilitation of greater severity and death of COVID-19 patients. In fact, Paital and Agrawal [196] demonstrated a link between NO_2_ emissions, PM_2.5_ levels, high ACE2 expression and COVID-19 infection severity. In summary, the mechanism may be attributed to air pollution-mediated co-morbidities, aerosol-induced respiratory disorders, and NO_2_-induced higher expression of ACE2 receptor that acts as a binding ligand for SARS-CoV-2 in respiratory cells in humans. Specific areas in India, China, Italy, Russia, Chile, and Qatar that experience heavy air pollution have also shown higher rates of COVID-19 infection and severity.

Furthermore, a “double-hit” hypothesis of the SARS-CoV-2 infection mechanisms and severe lung disease induced by the combined effect of PM_2.5_ and NO_2_ has been proposed by Frontera et al. [197]. In particular, ACE2 plays a bifunctional role as a sort of “double-edged sword”; it turns off the RAS and leads to beneficial effects but also mediates unique susceptibility to lung and cardiovascular disease in COVID-19 patients by serving as the SARS-CoV-2 receptor. Air pollutants (such as PM_2_._5_ and NO_2_) plus SARS-CoV-2 give a “double-hit” to the lungs leading to acute lung injury by attenuating tissue remodelling and influencing local inflammatory responses. Thus, chronic exposure to PM_2.5_ causes alveolar ACE2 receptor overexpression. This may increase viral load in patients exposed to pollutants, in turn depleting ACE2 receptors and impairing host defences. High atmospheric NO_2_ may provide a second hit causing a severe form of SARS-CoV-2 in ACE2 depleted lungs resulting in a worse outcome.

In summary, short-term, reactive virology-based measures (e.g., quarantines, repurposed drugs, etc.) are required to contain the present SARS-CoV-2 outbreak. However, the long-term, proactive toxicology-based measures required to intrinsically strengthen the immune system and prevent such future outbreaks need to be addressed [156].

Exposure to air pollution could increase vulnerability and have detrimental effects on the prognosis of patients affected by COVID-19. However, the relative weight of air pollution, compared to other confounders, is still to be determined by experimental and epidemiological studies, which are urgently needed for evaluating the role of atmospheric pollution in certain populations. Notably, the identification of vulnerable populations and continuous effort to lower air pollution ought to be critical steps.

**Table 1:** Summary of the main discussed findings underling the relationship between high levels of air pollutants and increased risk/fatality of SARS-CoV-2 infection.

Area of The Study	Size of Population	Air Pollutants	Effect on COVID-19	Reference
* China (for SARS-CoV-1)	>5000 cases of SARS-CoV-1 leading to nearly 350 fatalities	Air pollution evaluated by air pollution index (API)	In parts of China with moderate levels of air pollution, the risk of dying from the disease was >80% higher compared with areas with relatively clean air, and in heavily polluted regions the risk was twice as high	Cui et al., 2003 [178]
China	213 cities	Air pollution	Temporal increases in COVID-19 cases were associated with short-term variations in ambient air pollution	Zhu et al., 2020 [179]
China	120 cities	PM_2.5_, PM_10_, CO, NO_2_, and O_3_	A statistically significant relationship between short-term exposure to higher air pollutants (PM_2.5_, PM_10_, CO, NO_2_, O_3_) and increased risk of SARS-CoV-2 infection	Zhu et al., 2020 [179]
Italy	All Italian regions (*n* = 104,212 total number of cases)	NO_2_ and PM_2.5_	- Correlation between PM_2.5_ and COVID-19 outbreak distribution was observed - The highest number of COVID-19 cases were recorded in the most polluted regions, with patients presenting with more severe forms of the disease requiring ICU admission. In these regions, mortality was two-fold higher than the other regions despite similar rates of ICU admission (crude death rate 14% vs. 7%) - Chronic exposure to PM 2.5 caused alveolar ACE2 receptor overexpression	Frontera et al., 2020 [197]
Italy	Northern area: 71 provinces including Bergamo, Brescia, and Milan	Chronic exposure (PM_10_, PM_2.5_, NO_2_, and O_3_)	Higher mortality correlated with poor air quality, namely, with high PM_2.5_, NO_2_, and O_3_ values	Fattorini and Regoli 2020 [184] and Conticini et al., 2020 [185]
Italy	110 provinces	PM_2.5_	- There was a correlation between air pollution and the rate and outcome of SARS-CoV-2 infection. - Support the hypothesis that pollution-induced over-expression of ACE2 on human airways may favour SARS-CoV-2 infectivity	Borro et al., [163]
United States (USA)	A cross-sectional study nationwide	NO_2_, PM_2.5_, O_3_	There was an Estimated association between long-term (2010–2016) county-level exposures to NO_2_, PM_2.5_, and O_3_ and county-level COVID-19 case-fatality and mortality rates	Liang et al., 2020 [175]
USA	More than 3000 counties (representing 98% of the population)	Air pollutants—PM_2.5_	- Significant overlap between the causes of death in COVID-19 patients and those that lead to mortality from PM_2._5__ - An increase of 1 μg/m^3^ in PM_2.5_ was associated with an 8% increase in the COVID-19 mortality rate.	Pozzer et al., 2020 [162] and Wu et al., 2020 [186]
USA	From the environmental protection agency (EPA) website	O_3_, NO_2_, CO, and SO_2_	Ground-level O_3_ and NO_2_ concentrations contributed to a greater COVID-19 mortality rate	Liu and Li 2020 [161]
Several countries: China, Iran, Italy, Spain, France, Germany, the United Kingdom, and USA.	Both infections and deaths due to COVID-19 were collected and normalised by population size per administration unit (100,000 residents)	Associating several annual satellite and ground indexes of air quality (PM_10_, PM_2.5_, SO_2_, CO, NO_2_, and O_3_)	- Statistically significant positive correlations between COVID-19 infections and a high level of air pollution (long-term exposure) in each country was reported. - The higher mortality was correlated with poor air quality, namely, with high *PM_2.5_* and NO_2_ values	Pansini and Fornacca 2020 [182]
Italy and England	- Northern Italy - UK Biobank data (cohort of 1450 subjects)	NO_2_	NO_2_ correlated with mortality rates.	Ogen et al., 2020 [187], Travaglio et al., 2020 [189], and Filippini et al., 2021 [188]
Spain	372 of the 378 Basic Health Areas in Catalonia (population 371–72,321 inhabitants, mean 20,266)	NO_2_ and PM_10_	Association was found between long-term exposure to air pollutants and an increased risk of incidence and death from COVID-19: exposure to NO_2_ and, to a lesser extent PM_10_ were independent predictors of the spatial spread of COVID-19	Saez et al., 2020 [166]
Mexico	Metropolitan Mexico city. Pediatric and young adult onset of Alzheimer’s diseases	Nanoparticles (NPs)	In a worst-case scenario, SARS-CoV-2 and NPs may exacerbate the adverse health effects also on the central nervous system	Calderon-Garciduenas et al., 2020 [194]

* This study has been included as the first and high cited observation showing air pollution association and increased fatality of SARS patients in a Chinese population. Abbreviations: ICU, Intensive Care Unit.

A lesson from a substantial number of surveys and reviews on the environmental perspective of the COVID-19 pandemic is that the quest for effective policies to reduce anthropogenic emissions, which cause both air pollution and climate change, needs to be accelerated. The pandemic spread of the SARS-CoV2 ends with the vaccination of the population or with herd immunity through extensive infection of the population. However, since there are no vaccines against poor air quality and climate change, the remedy is to mitigate emissions. The transition to a green economy with clean, renewable energy sources will further both environmental and public health locally through improved air quality and globally by limiting climate change [162].

## 6. Conclusions

The comprehension of the mechanisms leading to the late stage of COVID-19 is imperative to manage the syndrome and to avoid/reduce the dangerous respiratory failure and the worst prognostic event. An imbalance between the protective and detrimental responses of the immune system drives severe symptoms and deterioration of patient conditions. The presence of chronic diseases characterised by high inflammatory mediator levels such as cardiovascular diseases, hypertension, diabetes, and obesity are pivotal factors for the development of severe COVID-19. Data from several places document an enhanced rate of both infection and severity in COVID-19 patients in polluted areas. Long-term exposure to air pollution increases the danger associated with four of the biggest COVID-19 mortality risks: diabetes, hypertension, coronary artery disease, and asthma. It also can make the immune system overreact, exaggerating the inflammatory response to common pathogens. Other differences between asymptomatic and moderate/severe patients are under evaluation, and some of them are related to genetic and metabolic factors.

Thus, COVID-19 represents an emerging pathological condition that has led researchers to re-evaluate the interactions of inflammatory and immune processes.
